# Evidence and Value: Impact on DEcisionMaking – the EVIDEM framework and potential applications

**DOI:** 10.1186/1472-6963-8-270

**Published:** 2008-12-22

**Authors:** Mireille M Goetghebeur, Monika Wagner, Hanane Khoury, Randy J Levitt, Lonny J Erickson, Donna Rindress

**Affiliations:** 1 BioMedCom Consultants inc, 1405 Transcanada Highway, suite 310, Dorval, Québec H9P 2V9, Canada; 2 Centre Hospitalier Universitaire de Montreal – McGill University Hospital center (CHUM-MUHC) Technology Assessment Unit, Montreal, Québec, Canada

## Abstract

**Background:**

Healthcare decisionmaking is a complex process relying on disparate types of evidence and value judgments. Our objectives for this study were to develop a practical framework to facilitate decisionmaking in terms of supporting the deliberative process, providing access to evidence, and enhancing the communication of decisions.

**Methods:**

Extensive analyses of the literature and of documented decisionmaking processes around the globe were performed to explore what steps are currently used to make decisions with respect to context (from evidence generation to communication of decision) and thought process (conceptual components of decisions). Needs and methodologies available to support decisionmaking were identified to lay the groundwork for the EVIDEM framework.

**Results:**

A framework was developed consisting of seven modules that can evolve over the life cycle of a healthcare intervention. Components of decision that could be quantified, i.e., intrinsic value of a healthcare intervention and quality of evidence available, were organized into matrices. A multicriteria decision analysis (MCDA) Value Matrix (VM) was developed to include the 15 quantifiable components that are currently considered in decisionmaking. A methodology to synthesize the evidence needed for each component of the VM was developed including electronic access to full text source documents. A Quality Matrix was designed to quantify three criteria of quality for the 12 types of evidence usually required by decisionmakers. An integrated system was developed to optimize data analysis, synthesis and validation by experts, compatible with a collaborative structure.

**Conclusion:**

The EVIDEM framework promotes transparent and efficient healthcare decisionmaking through systematic assessment and dissemination of the evidence and values on which decisions are based. It provides a collaborative framework that could connect all stakeholders and serve the healthcare community at local, national and international levels by allowing sharing of data, resources and values. Validation and further development is needed to explore the full potential of this approach.

## Background

The objective of any healthcare intervention is to improve health; preventive measures, non-pharmacological and pharmacological treatments, and medical procedures are among the numerous available options. Decisionmaking in healthcare is a complex process taking place along a continuum that moves from evidence generation to deliberation on each particular intervention and communication of the resultant decision.

Evidence-based medicine and evidence-informed health policymaking rely on evidence generated by developers of healthcare interventions, at least in the initial stages of the life cycle of an intervention. Evidence quantity, quality, usability and accessibility have been identified as hindrances to informed policymaking,[1] highlighting the disconnect between those who need evidence to make a decision and those who generate this evidence. Beyond evidence, decisionmaking requires value judgment. [2,3] Tunis argues that controversy around decisions may stem from the absence of shared views about the role of evidence versus judgment in evidence-based healthcare policies.[3]

Frequent controversy surrounding drug coverage variation across jurisdictions with similar levels of economic development, values and political systems [4-7] highlights a need for rational and transparent approaches to decisionmaking. Surveys have recognized a need for fair and explicit healthcare decisionmaking processes that are more defensible.[8,9]

Such processes should fulfill two main functions. Firstly, they should support the complex deliberative process that requires simultaneous consideration of multiple factors such as clinical benefit,[10] level of innovativeness,[6,10] quality of clinical evidence,[4,10] quality of dossier [i.e., organization, accuracy of information presented],[10] cost-effectiveness,[10,11] price and budget impact,[6,10] value judgments,[10] and colloquial evidence [anything that establishes a fact or gives reason for believing something].[12] Without an explicit process to structure such complex deliberation, decisionmakers are likely to resort to intuitive and subjective approaches, potentially missing important information.[13]

Secondly, such processes should help legitimize the decision by ensuring that conditions for 'accountability for reasonableness' (A4R) are met by structuring the deliberative process to make rationale and principles on which decisions are based explicit and ultimately publicly available. Within the A4R framework, availability to public scrutiny is a necessary prerequisite to legitimizing decisions.[14] As suggested by Dhalla and Laupacis, transparency in all areas of healthcare policymaking, including availability of data and decisionmaking rationales, is likely to raise public confidence in the process and may ultimately lead to better decisions.[15]

Several approaches have been published for making healthcare coverage decisionmaking more consistent, rational and transparent. [16-19] For example, the Cancer Care Ontario Policy Advisory Committee developed a tool that supports the deliberative process by presenting structured synthesized information on various aspects of the drugs considered.[17] A number of UK Health Authorities have developed explicit multicriteria models to facilitate prioritization decisions.[18] These attempts highlight growing awareness in those at the forefront of decisionmaking, and others in the field, of the need for a more holistic approach that goes beyond reliance on cost-effectiveness criteria. [20-23]

In this context, we hypothesized that healthcare decisionmaking could be facilitated by structuring access, consideration and communication of the evidence and the value judgments on which it is based. The objective of this study was to develop a practical framework to facilitate decisionmaking by supporting the deliberative process, permitting access to relevant evidence, and enhancing effective communication of decisions.

## Methods

Extensive analyses of the literature and of current decisionmaking processes were performed to identify steps leading to decisions, as well as the components of the thought processes underlying decisions. Needs and methodologies available to support such processes were identified to lay the groundwork on which to build the EVIDEM framework.

Review of decisionmaking processes in jurisdictions worldwide [10,24-44] was performed to explore the continuum from evidence generation to decision, to communication of decision. Processes for drug coverage decisions were used as a model since they are often the most structured and explicit in healthcare decisionmaking; however, all analyses were performed from the perspective of facilitating decisions for any type of healthcare intervention. Based on this review, the current steps flow as follows: (1) manufacturers/innovators generate data with experts and submit evidence to the decisionmaking body following specific requirements; (2) assessors & reviewers collect and appraise evidence (quality assessment), prepare a report for a decision committee (synthesized evidence) and may incorporate stakeholder opinion; (3) a committee makes a decision based on that report and stakeholder opinion; (4) the decision is made public with rationale for decision; an appeal process may be in place. Drawing from this analysis, the following needs were identified:

∘ systematic and explicit consideration of all key elements of decision during the deliberative process;[17,18]

∘ each committee member's perspective needs to be captured and values shared in the committee;[16,45]

∘ relevant evidence in a digested, unbiased and systematic format;[16,17]

∘ data on quality of evidence in a structured system for all types of evidence considered;[18] and

∘ transparent, understandable, and acceptable communication of decision.[15,41]

These needs were all considered in developing the framework. Analysis of the literature revealed that decisionmaking can broadly be subdivided into scientific judgment and value judgment.[2,3]

Scientific judgment relies on globally accepted standards defining the quality of evidence. Such technical judgment can be applied using a system in which the elements of quality are explicitly identified and quantified (scored). Scientific technical judgments are not highly dependent of the evaluator (compared to value judgments) and can be standardized. A number of quality standards, country specific guidelines, checklists and instruments are available to assess the quality of various types of evidence (e.g., CONSORT,[46] CHEC,[47] STROBE[48], QUOROM[49], MOOSE[50],. GRADE[51,52], QHES[53,54] and others [24-27,29-40,55-67]). While these provide a rigorous scientific basis for quality assessment of evidence, additional elements were identified that could integrate scientific judgment into a practical approach to healthcare decisionmaking. These include:

∘ streamlining quality assessment for all types of evidence;

∘ distinguishing between quality of reporting, and relevance and validity of evidence;

∘ providing the rationale behind scoring for full transparency; and

∘ using systematic deliberative processes to collaboratively evaluate the quality of evidence.

Analysis of the literature on quantifiable tools for value judgments considered in decisionmaking pointed to multicriteria decision analysis (MCDA). MCDA structures the deliberative process by breaking down a problem into the components expected to impact the value of an option, and by quantifying them using a scale with defined anchors.[13,68] MCDA explores value judgment from two standpoints: the value system of the evaluator with regard to the importance of each value components (weights) and the actual performance of an intervention (scores). A value estimate is obtained by combining weights and scores using simple or complex mathematical models. MCDA is widely used to support decisions in environmental engineering, agriculture, and marketing[13] and is a promising approach to healthcare decisionmaking [69-74].

Review of decisionmaking processes revealed that not all value components usually considered in decisionmaking are readily quantifiable.[10,24-44,61-67] A commonly shared direction of scoring is needed to define low and high ends of a scale to make quantification meaningful. In general, components defining the intrinsic value of an intervention are quantifiable from a universal standpoint, while extrinsic or system-related components are not readily quantifiable or quantification scales depend on specific local considerations. For example, when considering the intrinsic value component "improvement of efficacy", it is generally agreed that, all else being equal, an intervention that brings major efficacy improvement has a higher value than one with minor improvement. However, components such as historical context, stakeholder pressure, population priorities and access, and ability of a healthcare system to make appropriate use of intervention, factors often critical in healthcare decisions,[41,75,76] do not have consistent impact on how an intervention is valued. For these components, what constitutes increase or decrease in value requires definition during deliberation at the jurisdictional level and on a case-by-case basis. Consideration of extrinsic components is easier once intrinsic value components have been defined.

To facilitate value judgments related to a healthcare intervention, following needs were identified:

∘ disentangle intrinsic and extrinsic value components;[75,76]

∘ develop a simple and rigorous system that applies MCDA from a pragmatic standpoint based on actual thought processes;

∘ provide practical access to the evidence on which value judgments are based; and

∘ provide a practical method for decisionmakers to provide feedback to data producers and all other stakeholders.

Thus identified, these needs were used to develop the EVIDEM framework, processes and tools.

## Results

### Framework

A practical framework was developed structuring and making more shareable what is currently being done around the world. It was based on three main principles:

∘ Support deliberative process by disentangling and quantifying when possible scientific judgment (quality of evidence) and value judgment (intrinsic and extrinsic value of intervention);

∘ Facilitate access to relevant evidence over the life cycle of a healthcare intervention using a collaborative structure; and

∘ Enhance communication of decisions using transparent tools.

**Figure 1:**
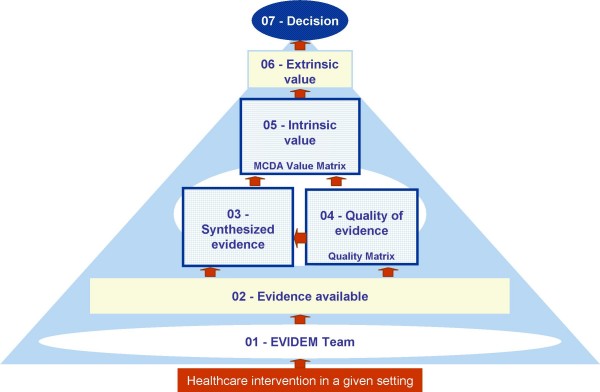
**Conceptual framework for healthcare decisionmaking in a given setting**.

The framework structures the context of decisions for a healthcare intervention in a given setting into seven modules (Figure 1). The centerpiece of the framework is the MCDA Value Matrix (module 5) which is both a quantification tool for the intrinsic value of an intervention and a portal to evidence (synthesized [module 3] with electronic links to full text source information [module 2]) and to data on quality of evidence (module 4 – Quality Matrix). Extrinsic value is considered in module 6 and communication of the decision is module 7. Applying the full framework from the early stage of development of a healthcare intervention requires a collaborative approach (module 1) in which all stakeholders are involved, i.e., decisionmakers, experts, data assessors and data producers. The result of the process is an EVIDEM record, modules of which can be shared in a web-based collaborative database for transparency and application by other decisionmaking groups or individuals. The modular aspect facilitates access to evidence and decisions, updates, and database development.

### Value of intervention – Value Matrix

A MCDA Value Matrix (VM) was developed to include the value components usually considered in policy decisionmaking. MCDA was selected as a methodological model for the VM for its versatility, transparency and ease of application by a wide range of stakeholders. Value components that can not be readily incorporated into a matrix were not included and were listed as extrinsic components for consideration at the jurisdictional level or on a case-by-case basis (e.g., equity, historical context, stakeholder pressure, population priorities and access, ability of healthcare system to make appropriate use of intervention) (module 6).

The VM (module 5) was designed to address the key question: What is the value of a healthcare intervention with respect to its intrinsic characteristics? In other words, what does it bring to the health of society (for the jurisdiction being considered)? Such a question involves probing the value system of decisionmakers (weights) and assessing the healthcare intervention based on evidence available using defined scales (scores). The value estimate of an intervention is the combination of weights and scores.

Components of decisionmaking identified in the analysis of current decisionmaking processes were specifically defined and structured to fulfill MCDA methodological requirements.[68] These are:

• Completeness: all currently-understood components defining the intrinsic value of an intervention are included;

• Non-redundancy: all components are necessary, important and there are no duplicates;

• Mutual independence: scoring of each component is independent from scoring of all other components (i.e., scores for each component can be assigned without considering scores for other components); and

• Operationality: each component is defined unambiguously; data on which to base the evaluation is available; the numerical scale follows a shared sense of direction.

**Figure 2:**
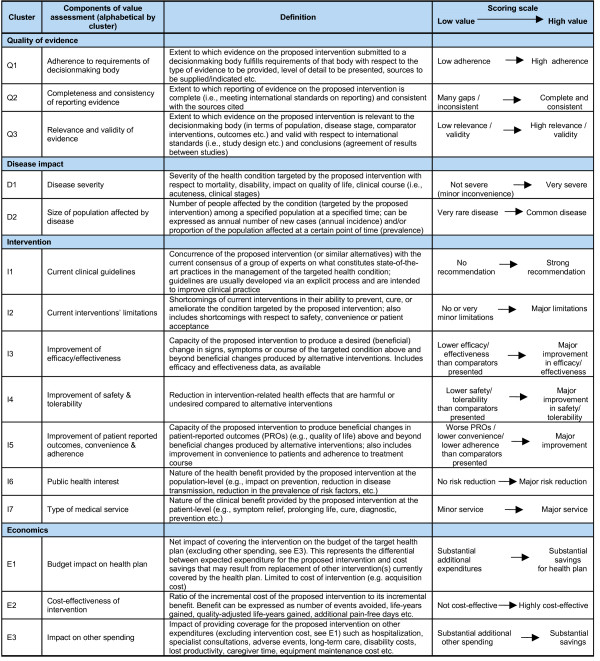
**Value Matrix – definitions of components and scoring scales**.

Fifteen components were thus defined and grouped into four clusters; scoring directions were defined from a societal perspective (Figure 2)[68]. The first cluster assesses the impact of the quality of evidence on the value of an intervention (e.g., how the relevance and validity of evidence impacts the value of an intervention). This is not to be confused with the assessment of the quality of evidence, which is performed separately using the Quality Matrix (QM, see below). One key principle of EVIDEM is that reasoning is facilitated and made more objective by disentangling these distinct concepts (quality of evidence based on scientific standards versus the value assigned to the quality of evidence). The first cluster was broken down into three components corresponding to the three criteria of the QM.

The disease impact cluster was broken down into two components: disease severity (D1) and size of affected population (D2). It was assumed that an intervention for a very severe disease has more value than an intervention for a mild disease (D1) and that an intervention that benefits a large number of patients has more value than one that benefits a small number of patients (D2).

The intervention cluster was broken down into seven components. The first (I1) explores the impact of clinical guidelines. Clinical guidelines serve multiple functions for numerous groups and have become ubiquitous.[77] They can have considerable impact on practice and perceived value of an intervention.[78] It was assumed that guidelines represent current consensus and that strong (e.g., Class I)[79] recommendations for the intervention under consideration or for a similar intervention (e.g., a product structurally related[80]) would result in a high value score. The second component assesses the impact of limitations of current interventions (I2) on the value of a new intervention.

The concept of improvement of medical service, used by the Commission de la Transparence in France,[81] was used to define three key components of the value of an intervention: efficacy and effectiveness (I3); safety and tolerability (I4); and patient reported outcomes, convenience and adherence (I5). Assessing these components required clearly defining which existing medical services and medical practices the new treatment is meant to replace or complement. Data for these existing services provides the evaluator with an evidence-based frame of reference for components I3 to I5. Components I6 "Public health interest" and I7 "Type of medical service" capture the nature of the health benefit of the intervention respectively at the population level and at the individual level.

The economics cluster was broken down into three components to explore the impact of covering a new intervention on health plan budgets (E1), on other spending (E3), and its cost-effectiveness (E2). To ensure non-redundancy and to be in line with standard budget impact modeling practices, components E1 and E3 respectively were defined to cover financial impact of intervention only (limited to the cost of intervention and potential savings in replacement of existing interventions) and all other economic impacts (such as those resulting from changes in hospitalization, adverse events, disability, equipment maintenance cost). The latter is usually explored in economic evaluations, which are made more useful to decisionmakers by reporting disaggregated cost-consequence information.[82] The economic evaluation component (E2) assesses the value of an intervention based on cost-effectiveness ratios obtained from the analytic perspectives (e.g., healthcare system, societal). Although this component is partly redundant with several VM components, it was included in the VM to reflect current decisionmaking practices.

**Figure 3:**
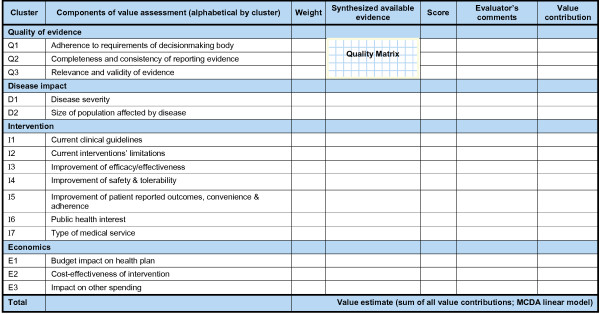
**Value Matrix – assessment of the intrinsic value of a healthcare intervention**.

The VM was then designed to be self-contained, with an emphasis on practicality (Figure 3). It contains:

• A **weighting scale **(1 to 5) to capture the value system of each evaluator independent of the healthcare intervention under scrutiny; standard deviation of weights for each VM component (W_x_) among a group of evaluators can be used to support discussion among evaluators;

• **Synthesized evidence **for the healthcare intervention under scrutiny prepared using a standardized methodology to minimize bias (see below);

• A **scoring scale **(0 to 3) with defined anchors and scoring guidelines; it includes four scoring options to stimulate thought processes and avoid loss of information with a middle score, and zero to allow for exclusion of a component that does not bring any value (e.g., safety less than current practice); standard deviation of scores for each VM component (S_x_) can further stimulate deliberative process among evaluators;

• A **comments section **for decisionmakers to provide feedback to the producers of evidence; includes a prompt to indicate whether low score is due to data limitation, providing a way to capture and communicate data needs;

• A **simple MCDA linear model **to capture a value estimate (V) of the intervention for each evaluator:

$$\text{V}=\sum_{x=1}^{\text{n}}{\text{V}}_{x}=\sum_{x=1}^{n}\left(\frac{{\text{W}}_{x}}{\sum W_{n}}{\text{S}}_{x}\right)$$

Where

W_x _is the weight for each VM component

S_x _is the score for each VM component

∑W_n _is the sum of all weights (i.e., for all *n *VM components)

V_x _is the value contribution for each VM component

**Figure 4:**
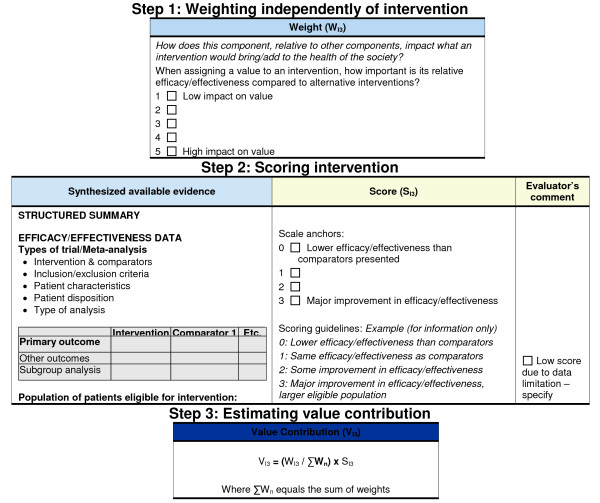
**Value Matrix – assessing Improvement of efficacy/effectiveness (component I3)**.

An example of how a VM component is assessed is shown in Figure 4 (I3: Improvement of efficacy/effectiveness).

**Figure 5:**
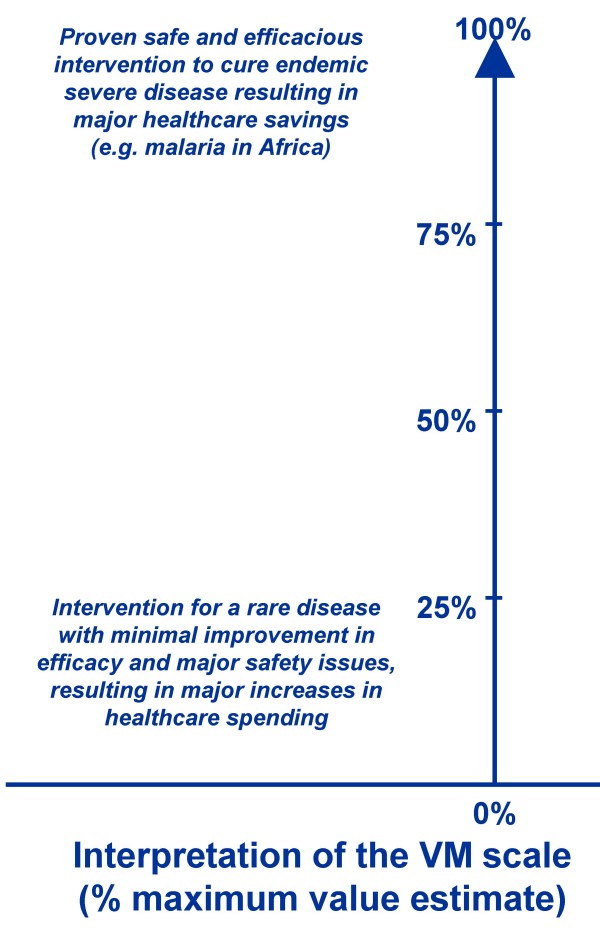
**Value Matrix comparative scale**.

The value estimate of a healthcare intervention obtained from an individual or from a group of evaluators is reported in the VM Comparative Scale as a percentage of maximum score allowing for comparison across healthcare interventions (Figure 5). Interpretation of results requires clear understanding of the meaning of the value estimate, including its maximum and minimum anchors. Anchors incorporate all the dimensions captured by the components of the VM, thus providing a broad scale for valuing all types of interventions. Because some components of the VM are time bound (e.g. improvement of efficacy over existing intervention at a point in time), the value estimate will change over the life cycle of the intervention as new interventions are made available.

### Access to evidence – synthesized and full text

Access to high level synthesized evidence is necessary to focus the thought process on key elements of decision but should be complemented by easy access to full text sources for those who want to access more details.

To ensure minimally biased evidence is available to stakeholders, a methodology was developed to synthesize this evidence for each component of the VM (module 3). The principal objective was to provide the information necessary and sufficient to score each component with access as needed to full text sources. A template with instructions was developed for each component of the VM indicating where and how to find evidence (search algorithms, biomedical and economic databases, registries, manufacturer, health technology assessment reports, Cochrane reviews, etc.), what to report and how (i.e., standard format). For full traceability, electronic links to full text sources were integrated into module 2.

For example, to assess "disease severity", data to be identified and reported included disease acuteness, morbidity (disability, quality of life) and mortality, as well as disease stages or subtypes that differentiate therapies and target populations. Besides extracting study results, key elements used to define their validity are also reported, such as, number of patients included in pivotal trials, follow-up duration for safety data, key model features for economic evaluations and sources used for budget impact projections. For the quality of evidence cluster, quality scores for each type of evidence are provided by criterion of quality assessment (quality scores are obtained via an explicit process described below), providing decisionmakers with structured access to results and rationale of quality assessment for each type of evidence.

### Quality of evidence – Quality Matrix

**Figure 6:**
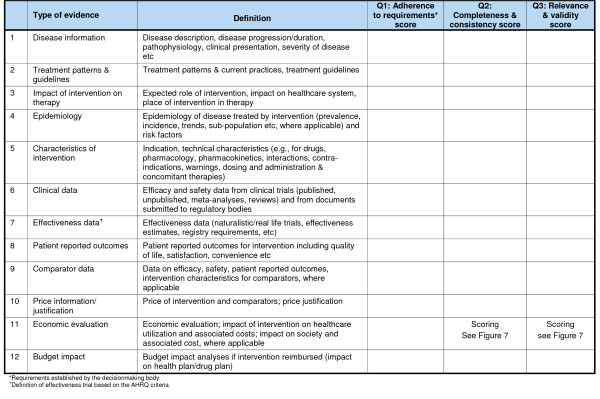
**Quality Matrix – assessment of quality of evidence for a healthcare intervention**.

The QM was designed to quantify the quality of evidence generated for a healthcare intervention; it is grounded in current evidentiary requirements of healthcare decisionmaking bodies and derives from numerous existing tools and instruments to assess quality of evidence. The QM streamlines quality assessment of all types of evidence, disentangles criteria of quality, and provides access to a rationale for each score attributed via a deliberative process. The QM was designed with an emphasis on practicality and includes (Figure 6):

• Three criteria of quality assessment (columns);

• 12 types of evidence currently required (rows);

• For each cell of the QM:

∘ Questions or instruments based on global standards

∘ Prompt for evaluator to provide rationale for score

∘ Scoring scales

Five elements defining quality were identified and clustered in three criteria (Figure 6):

• **Q1 Adherence to the requirements **established by the decisionmaking body to which evidence is submitted;

• **Q2: Completeness **of reporting, as prescribed by reporting guidelines, and **consistency **with cited sources and throughout the document; this criterion can be applied to individual studies or to a high-level document (e.g., dossier) that includes several studies;

• **Q3: Relevance **of evidence to the decisionmaking body and **validity **of evidence, with respect to scientific standards and methodological guidelines in applicable fields of research.

Selection of the types of evidence (12 rows of the QM, each representing a research field, such as clinical research, health economics, epidemiology, pathology) for inclusion in the QM was based on an analysis of current evidentiary requirements of over 20 decisionmaking bodies worldwide [24-28,30-40,42-44,76] ensuring that all essential requirements were covered. This analysis also permitted creating definitions for each type of evidence that were sufficiently detailed to standardize QM use and support cross-jurisdictional comparability (Figure 6).

Evidence concerning the disease and its management was broken down into three types: disease description (#1), current treatment patterns including practices and guidelines (#2), and impact of new intervention on therapy (#3). Epidemiology data included standard metrics and risk factors (#4). Information on the new intervention was broken down into four types: characteristics of intervention (#5), efficacy and safety data obtained from clinical trials (#6), patient reported outcomes (PRO) data (#7) and effectiveness data from trials and registries (#8) For the last type of evidence, identification of effectiveness used the criteria defined by the US Agency for Healthcare Research and Quality (AHRQ).[83] Data on current interventions that the new intervention is projected to replace or complement was captured in a separate component (Comparator intervention data # 9) including efficacy, safety, PRO and effectiveness data, and characteristics. Economic data was broken down into three types of evidence: price and price justification (#10); economic evaluation including impact of the new intervention on healthcare utilization and costs, and on society (#11); and impact of reimbursing the new intervention on the health plan budget (#12).

**Figure 7:**
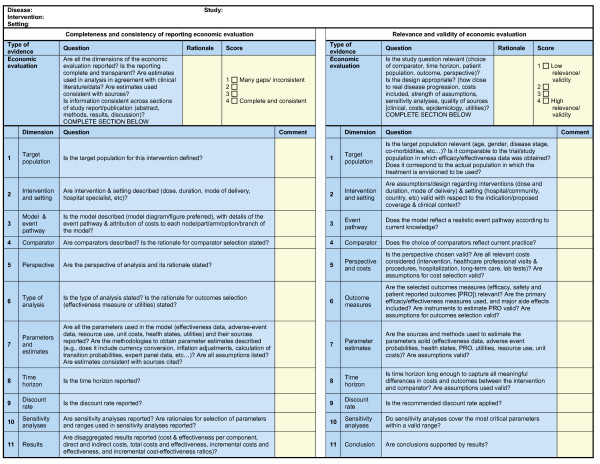
**Quality Matrix – assessing quality of economic evaluations**.

For each type of evidence contained in the QM, instructions, questions, and for the most complex types of evidence, specific instruments were developed. They were derived from current tools (e.g., GRADE, CHEC, etc.) to streamline scoring processes across types of evidence, distinguishing criteria of quality (e.g., reporting versus validity) while keeping the whole system practical. For example, for type of evidence "Economic evaluation", two 11-dimension instruments were developed: 1) an instrument to assess the completeness and consistency of reporting of the study; and 2) an instrument to assess the relevance and validity of study design and results (Figure 7).

A scoring scale with defined anchors was developed and full transparency requires that each score be justified by the investigator. Rationale and scores are reviewed by another investigator and validated by experts through deliberative process until consensus is reached. Comments, rationale and scores are all integrated into the QM for full traceability. Aggregated quality scores are estimated as a percentage of maximum score by criterion, by type of evidence or for the whole QM.

## Discussion

The EVIDEM framework was tailored to reflect the thought process underlying decisionmaking and to fit the continuum from data generation to decision to communication of decision. It supports decisionmaking and deliberative processes by structuring, segregating and providing transparent access to evidence (incorporating quality assessment), while facilitating communication about value judgments and data needs among stakeholders.

The instruments developed to operationalize the EVIDEM framework are rooted in existing processes and instruments; however, they integrate the essential components of decisionmaking into a comprehensive and cohesive structure. The VM draws on the flexibility and comprehensiveness of MCDA while disentangling extrinsic from intrinsic value components, and providing structured access to the evidence on which those value judgments are based. Unlike some earlier applications of MCDA,[69,74] the VM does not require complicated mathematical models or computation, but rather serves as a communication tool among and between stakeholders. Specific instruments developed for the QM draw on existing instruments in each respective field of research. These often combine in one instrument dimensions pertaining to quality of reporting and to relevance and validity of a study (e.g., for economic evaluations[47,54,56]). QM instruments disentangle quality of reporting (Q2 completeness and consistency) from relevance and validity (Q3), requiring the reviewer to focus specifically on each aspect of quality, bearing in mind that relevance and validity require good reporting practices to be fully evaluated. Because results of quality assessment are highly dependent on the assessor, rather then on the instrument, it was suggested by Gerkens et al,[84] that assessors should reach a consensus on scores, which is required when applying the QM instruments.

The EVIDEM framework needs to be tested in context, validated and further developed through iterative collaborative processes. In a proof of concept approach, the system was pilot-tested using historical cases in the Canadian context with the objective of assessing feasibility, practicality and value to end users. The Canadian Value Panel convened for the pilot study indicated that the VM with embedded synthesized data would be highly useful as a support for healthcare decisionmaking, to guide discussion and share values among decisionmakers, at both the policy and clinical levels, by systematically assessing strengths and weaknesses of healthcare interventions in a comprehensive and structured fashion.

Practical use of this approach faces significant challenges. Among these are uptake by decisionmaking bodies; this will only happen if the new process is perceived as facilitating and simplifying their task, rather than adding complexity. The EVIDEM framework was designed to create a simple and practicable series of freely accessible tools that could be easily integrated into existing processes, while providing a common ground. In addition, integration of EVIDEM records into a web-based collaborative database is intended to provide a platform to all stakeholders for easy access to high level data on evidence available for healthcare interventions, as well as to value estimates.

Another major challenge will be the bringing together of data producers and those who make decisions. There are issues of trust and bias that need to be surmounted to provide the collaborative environment that this process would need. This would permit the 360 degree transparency as envisioned by Dhalla & Laupacis.[15]

**Figure 8:**
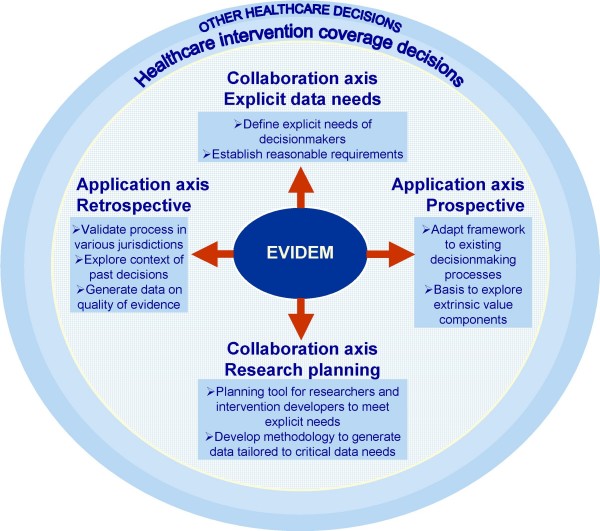
**Potential applications of the EVIDEM framework**.

The framework was designed to be of use to a variety of healthcare decisionmakers. Several applications are envisioned (Figure 8). Retrospectively, the approach can be used to explore the context of past decisions, assess the quality of evidence available for a healthcare intervention at a point in time, and validate the process in a given jurisdiction (Figure 8 – Application axis). Prospectively, it can be used to evaluate new interventions and to maintain a transparent record of evidence and decisions over its entire the life cycle. Several studies assessing healthcare decisionmaking processes in various regions of the world have highlighted the importance of transparency and fairness.[8,85,86] A number of initiatives have been implemented globally to increase transparency in access to both evidence and rationale for policy decisions. In Canada, the Common Drug Review recently implemented a transparency initiative.[87] while in the UK, the National Institute for Excellence is now providing full access to manufacturer dossiers on their web site.[88] However, current processes for coverage decisions are generally organized in such a way that decision rationales cannot easily be shared among members of the decision committee, let alone members of the public. Using an approach such as EVIDEM to make and communicate decisions could represent a significant step towards a more accountable and transparent process. Better understanding of the rationale behind decisions by all stakeholders could in turn enhance the legitimacy and acceptability of decisions.[5,14,15] Similar reasoning could apply to decisionmaking at the individual level; patients and their healthcare team could use such an explicit framework to assist consideration of all the components of complex decisions.

Another aspect of healthcare decisionmaking, which requires further development, is extrinsic or system-related value judgments (Figure 8 – application axis). These may be critical in decisions and require focused discussion and elicitation of preferences or consensus building at the jurisdictional level. One study applying an MCDA approach to healthcare priority-setting in Ghana identified extrinsic factors such as 'age of target group' and 'poverty reduction' as critical factors through discussion with stakeholders and local policymakers.[72] Research in this area is essential to identify and structure system-related factors in decisions, which will be easier if predicated on transparent assessment of the intrinsic value of interventions.

Several features are integrated in the EVIDEM framework to facilitate communication between those who generate data and those who need data to make decisions. Through iterative processes, the framework can help define evidentiary needs of decisionmakers and be used as a planning tool for researchers and developers of new interventions, to ensure that the data that is generated addresses the needs explicitly defined (Figure 8 – Collaborative axis). Knowledge transfer and exchange (KTE), an interactive process between research users and research producers, aims to increase the likelihood that evidence will be used in practice and policy decisions.[89] A recent review suggests that this field of research, still in its infancy, has yet to identify KTE strategies that best support health policy decisionmaking.[89] Finally, the EVIDEM framework can also be used for educational purposes to explore the thought processes underlying healthcare decisionmaking and the concepts that define quality of evidence.

## Conclusion

Healthcare decisions have to be made in the context of a plethora of information, without easy access to all the necessary information and without an explicit decisionmaking framework. This often results in poor transparency and controversial decisions. The EVIDEM framework provides a comprehensive transparent structure grounded in global standards and local needs. The proposed framework is a step to organizing evidence and streamlining processes on a collaborative approach. This framework should not be viewed as a formula but rather as an aid to ensuring that all important data is considered and that rationales and values underlying a decision may be shared. It supports deliberative processes [12,90] allowing decisionmakers to combine all types of evidence and values, and increases the likelihood of making solid decisions. Validation and further development through collaborative and synergistic efforts is necessary to explore the value of this framework in practice. This type of systematized and shareable approach for data access and value assessment is expected to help optimize decisions, resources, and health.

## Abbreviations

AHRQ: US Agency for Healthcare Research and Quality; CHEC: Consensus on Health Economic Criteria; CONSORT: Consolidated Standards of Reporting Trials; EVIDEM: Evidence and Value: Impact on DEcisionMaking; KTE: Knowledge transfer and exchange; MCDA: MultiCriteria Decision Analysis; MOOSE: Meta-analysis Of Observational Studies in Epidemiology; PRO: Patient reported outcomes; QoL: Quality of life; QUOROM: Quality of Reporting of Meta-analyses; STROBE: STrengthening the Reporting of OBservational studies in Epidemiology.

## Competing interests

The authors declare that they have no competing interests.

## Authors' contributions

MMG, DR & MW conceived the framework, developed the instruments and drafted the manuscript. HK, RL and LJE participated in the design of the methodology and drafting of the manuscript. All authors read and approved the final manuscript.

## Pre-publication history

The pre-publication history for this paper can be accessed here:


